# COVID-19 Pandemic: Socio-Economic Consequences of Social Distancing Measures in Italy

**DOI:** 10.3389/fsoc.2020.575791

**Published:** 2020-10-16

**Authors:** Vincenzo Auriemma, Chiara Iannaccone

**Affiliations:** Department of Political and Social Studies, Sociology, University of Salerno, Fisciano, Italy

**Keywords:** coronavirus, digital divide, economy, empathy, social distance, work

## Abstract

The lock-down measures adopted in all countries of the world have led to far-reaching social and economic changes. The health emergency had immediate repercussions first on the social system and then on the economic one. The social repression measures taken to limit the infection have generated a drastic change in daily life, detaching ourselves from the other emotionally and physically. The already difficult situation that Italy was experiencing from an economic and social point of view is immediately exposed by the health emergency, and then worsened and extended to all sectors. In this context, it is important to study different types of phenomena: the suspension of commercial activities and the consequent repercussions on the work sector, smart-working and infrastructural and cultural digital divide, the new forms of interaction and relationship that transform the emotions and, finally, the enormous fluctuation of world markets. To face such a far-reaching crisis, the measures taken not only at national level, but also supranational and international will be decisive.

## Introduction

On January 30, 2020, China reported to the world the existence in the city of Wuhan of a cluster of cases of pneumonia of unknown etiology (later identified as a new coronavirus Sars-CoV-2), the same day the WHO (World Health Organization) declared the international state of emergency. The following day, January 31, the Italian government proclaimed a state of emergency and implemented the first measures to contain the infection throughout the national territory. One of the first measures adopted was the suspension of all flights to and from China with the implementation of airport controls, using thermoscanners for measuring body temperature, in order to monitor the health conditions of passengers from China through stopovers intermediate. The increase in health checks was immediately foreseen also in ports, involving not only non-EU boats but all merchant and cruise boats in transit on the national territory. On February 21, the Ministry of Health introduced mandatory quarantine isolation measures for close contacts with a case that tested positive for Covid-19, and ordered active surveillance with fiduciary home stay for those who were in risk areas in the last 14 days, with the obligation of reporting by the interested party to the local health authorities. On 23 February, following the outbreaks registered in Lombardy and Veneto, the first “red zone” was established, some municipalities were isolated (ban on expulsion and ban on access) and were suspended in them all educational and cultural activities, economic, commercial and recreational-recreational activities guaranteeing citizenship access to essential services and goods. On February 25, some measures to contain the contagion, concerning the suspension of sporting events, school activities and higher education (recommending the implementation of distance learning), cultural and tourism activities and driving exams, were extended to all the municipalities of the Regions: Emilia-Romagna, Friuli Venezia Giulia, Lombardy, Veneto, Liguria and Piedmont. On 1 March the same Regions entered the “red zone.” On 4 March, educational activities in schools and universities, congress activities, cultural events and sporting events were suspended throughout the country, recommending the use of agile work. On 8 March, a single containment area was created with more stringent measures including the territory of the Lombardy Region and 14 other Provinces (five from Emilia-Romagna, five from Piedmont, three from Veneto and one from Marche). With the DCPM (Presidential Decree of the Council of Ministers) 8 March, respect for the interpersonal safety distance of at least one meter is introduced for the first time as a containment measure and the need to avoid gatherings is highlighted several times, the use of the mask is instead recommended only to those who suspect to be sick and to those who care for sick people^1^. On 9 March the “red zone” is extended to the whole national territory and will remain in force until 4 May.

Maintaining social distance seems to be the most effective health device for preventing Covid-19 contagion. Since the coronavirus began its diffusion, two types of distances were recognized: one refers to the distance between individuals (gatherings) and the other to the distance that each individual must keep on the other, in order to avoid contagion (Bignami, 2020; CDC, 2020; Demarais, 2020). Social distance, sociology and social psychology, mean the willingness of members of one group to have social contacts with people from another group. In particular, it is studied in which way people are ready to exclude or admit those who belong to another group (Canavese, 2020). It is important to know that this measure is not new, its first application, even if not in these terms (1.8 million), dates back to September 1918, toward the end of the First World War, when a bad influence began to spread throughout the world. The virus responsible for the disease, which became known as Spanish flu, infected over a quarter of the world's population, with an estimated death toll of 50 to 100 million, it became one of the deadliest pandemics in human history. The first prohibitions and the first “social distances,” although they did not use this term correctly, began in the United States. At that time, some cities were organizing parades to promote ties of freedom, with the aim of helping to pay for the war efforts in Europe. In Philadelphia, Pennsylvania, where 600 soldiers had already been infected with the flu virus, a never-adopted rule was introduced, quarantine and social distance within the family system (Pottinger, 2013; Bourouiba, 2020; Carlini, 2020; Resnick, 2020).

The objective of this work is to investigate the consequences produced by the measures adopted by the Italian government, and subsequently by many other European and non-European countries, to deal with the Covid-19 pandemic starting from an analysis of the situation prior to the crisis and investigating some of the possible future scenarios. As is well-known, Italy suffers from various structural deficiencies made even more acute in the last decade by the economic policy recipes adopted for the restructuring of the exponential national public debt. The Covid-19 emergency had the merit, or demerit, of unmasking the deficits that have existed in Italy for years in all sectors of activity, public and private, and urgently imposes the need to remedy dramatically chronic situations, the result of political and economic actions. The importance of well-being, the importance of income and the critical issues raised by ever more marked inequalities have been rediscovered.

## The Consequences of Social Distances

### Economy and Work

The Istat (National Institute of Statistics) note on employed and unemployed of February 2020 shows a stable employment rate at 58.9%, the result of a slight increase in employment among women (+12 thousand units), temporary employees (+14 thousand) and young people aged 15 age 24 (+35 thousand) and a drop in employment among men (−22 thousand units), permanent employees (−20 thousand), self-employed persons (−4 thousand), and over 35 (−44 thousand) (Istituto Nazionale di Statistica, 2020b). The inactivity rate stands at 34.5% with an increase in February of women and people aged at least 35 equal to 12 thousand units. In the same month, women seeking employment (−39 thousand units) and the over 35s decreased, while men (+22 thousand units) and young people between 15 and 24 years increased. Unemployment stood at 9.7%, with a slight decrease of 0.1%, while youth unemployment remained stable at 29.6%. Comparing the December 2019–February 2020 quarter with the previous one, September–November 2019, there is a clear decline in employment (−89 thousand units) involving both gender components between 15 and 49 years, permanent employees and self-employed while it sees a slight growth among temporary employees. In the same quarter, the number of people seeking employment decreased and the inactive increased (+51 thousand units) (Istituto Nazionale di Statistica, 2020b). A not at all rosy pre-crisis picture that substantially records an increase in precarious work, a strong mistrust in the future and a dramatic youth condition. An even less comforting situation if you look at the latest data made available by the 2019 statistical yearbook on poverty (Istituto Nazionale di Statistica, 2019a,b,c). In 2018 the percentage of families in absolute poverty in Italy was 7% (882 thousand) with an alarming incidence in the South where the value stood at 20.5% (higher than the national average of 19.4%) (Istituto Nazionale di Statistica, 2019a,b,c). The highest incidence is recorded among families with five and more components (19.6%), followed by couples with three or more children (16.6%), single-parent families (11.4%) and families with four components (8.9%). The lowest incidence is recorded among families of and with the elderly (4%) and further decreases in families in which the reference person is over 64 years old (3.2%). On an individual level 8.4% of the entire population is in conditions of absolute poverty, of the 5 million and 40 thousand individuals in this condition over 2 million and 300 thousand reside in the South (11.4%) and over 2 million and 500 thousand are women (8.3%). The incidence of absolute poverty is high among minors (12.6%) and people aged between 18 and 34 (10.3%), confirming its minimum among over 74 years (4.6%) (Ibid). The elderly population in Italy proves to be the main social safety net, a figure that should not be underestimated if we consider that most of the deaths from Covid-19 belong to the age group between 60 and 90 years of age and over, on April 13, 2020 the number of deaths between 60 and 69 years is 11.5% of the total, between 70 and 79 years at 31.5%, between 80 and 89 years at 40.4% and among people over 90 years of age 11.6% (Il Sole 24 Ore., 2020).

Decisions on adopting social distance have inevitably involved almost all productive activities. In Italy, according to the ISTAT note on the economic trend of the month of March, the activities of 2.2 million companies, 49% of the total, have been suspended, investing the exporting companies to a greater extent, involving 65% of the total. The blockade of production activities involved 44.3% of employees and 42.1% of employees. The first response recorded by the National Statistical Institute is a sharp collapse in consumer and business confidence (Istituto Nazionale di Statistica, 2020a). In addition to the direct effects connected with the suspension of the work activities, the production sector also suffers from the indirect effect related to the cross-sectoral relations. An example is given by the expenses for fuels and land transport (for example bus) services which have fallen sharply and the expenses for tourism which have been completely eliminated. The first estimate of the effects of the blockade on ISTAT'S economic performance is not reassuring. In this situation, two types of scenarios have been hypothesized. The first one is related to the possibility that the limitation of production activities is limited only for the months of March and April, a reduction in final consumption of 4.1% is estimated, with a decrease in value added generated by the production system of 1.9%, involving 385 thousand employees, of which 49 thousand irregular, for an amount of approximately 9 billion euros in salaries. The most expensive price of the drop in value added is paid by the accommodation and restaurant services (−11.3%) and by the commerce, transport and logistics sectors (−2.7%), while the consequences on the less incisive sectors they are: producing investment goods and construction (less than one percentage point) (Istituto Nazionale di Statistica, 2020a).

The second one concerns the extension of the measures to the months of May and June, leading the reduction in consumption of 9.9%, with an overall reduction in added value of 4.5%, involving 900 thousand employees, of whom 103 thousand non-regular, for a total of 20.8 billion euros in salaries. Also, in this case the most marked contractions of the added value would involve the restaurant and restaurant business (−23.9%), trade, transport and logistics (−6.9%) with more marked effects on the production of consumer goods (−3.6%), personal services (−3.6%) and professional services (-3.4%). In this scenario, commercial services and “socialization” would pay the most expensive price, with a drop in value added of −16.4% in the cultural sector, −12.7% in the entertainment sector and −6.7% in the retail trade, potentially affecting 608 thousand employees, of whom 72 thousand are not regular (Istituto Nazionale di Statistica, 2020a).

In March, there was a slowdown in inflation attributable, according to ISTAT, to the slowdown in unregulated energy goods (−2.7%) and services (from + 1% to + 0.6%), these trends of decreases were only partially offset by the acceleration in food prices (from + 0.4% to + 1.2%) and tobaccos (from + 1.5% to + 2.5%) (Istituto Nazionale di Statistica, 2020a).

To date it is still difficult to estimate the number of people who, due to the Covid-19 emergency, could find themselves out of a job as it is difficult to predict the number of small and medium-sized enterprises that will be able to resist and get to phase 2, considering that many of them are already suffering. For now, a number of workers at risk of 10 million is assumed, based on this estimate the State has made 10 billion available with a bonus of 600 euros for 5.3 million workers who will reach 800 in April and May, while for the precarious, who demonstrate that they have worked at least 4 weeks a year, 400–500 euros per month were paid (Livelli, 2020; Mondani, 2020). An insufficient measure if we consider all those precarious workers of the informal economy which in the South amount to about 50%. Law Decree April 8, 2020, n. 23 (Urgent measures regarding access to credit and tax obligations for businesses, special powers in strategic sectors, as well as interventions in the field of health and work, extension of administrative and procedural conditions) “Credito” has disbursed € 400 billion in addition to the 350 billion already allocated by the decree 18/2020 “Cura Italia”, 200 billion were allocated to give liquidity to companies for those involved in the internal market and for those dedicated to exports. Again, the measures may be insufficient, in addition to the loans, non-repayable loans have not been disbursed (Istituto Nazionale di Statistica, 2020a).

The economic and financial crisis generated by this health emergency could have even more damaging consequences than the recent 2008 subprime mortgage crisis, because every new coronavirus outbreak in the world breaks the chains of a production system that is now strictly close interconnected globally. This is a shock that affects both demand and supply simultaneously and could cause generalized declines in production and supplies together with a recovery in inflation (Istituto Nazionale di Statistica, 2020a). Humanity is facing an unprecedented global crisis which, unlike the barriers raised by nationalist policies, has no borders. According to the International Monetary Fund (2020), 3% of world GDP (Gross Domestic Product) will increase in 2020, with economic losses of about 9,000 billion dollars, indiscriminately affecting rich and poor countries. Global supply chains have major flaws and the financial crisis involves markets and raw materials; advanced economies will pay the most expensive price with a contraction of −6% of GDP while for emerging countries it will be equal to 1%. The Eurozone will lose 7.5% of GDP, the taillight, immediately after Greece, Italy is for which a contraction of −9.1% is expected (Istituto per gli Studi di Politica Internazionale, 2020). In presenting the reports on the world economy in April 2020, the director of International Monetary Fund, Kristalina Georgieva, said that the ongoing crisis will lead to the worst economic fallout of the Great Depression, if an increase in per capita income was expected 3 months ago in over 160 member countries of the negative growth of the IMF is now forecast for 170 countries (Istituto Nazionale di Statistica, 2020a). Angel Gurria, OECD secretary general, said that in the event that countries manage to respond promptly to the shock and with appropriate measures, the recovery curve will resemble a U, with a long period of suffering that will last for years, otherwise it could become an L (Szu, 2020). This crisis presents several peculiarities, first of all the uncertainty of its duration and intensity, which is still impossible to define today, and also the impossibility of giving impetus to the economy with the usual measures, how to stimulate aggregate demand, where these they are undesirable for those sectors, most affected in compliance with containment measures (International Monetary Fund, 2020). As stated by Gita Gopinath, IMF economic consultant, this crisis must be faced in two phases: one of containment and stabilization and the other of recovery. In the first phase, blocking and social distancing measures are essential to slow down the transmission of the virus and give the health system time to expand its services and try to develop a vaccine through the right flow of resources. At the same time, it is essential that states implement the fiscal, monetary and financial measures necessary to keep the company's economic infrastructure intact. To reduce systemic stress, liquidity must be introduced to counteract loss of confidence and strengthen expectations for economic recovery. Governments and central banks will have to take a leading role in economies with the support of international financial institutions and bilateral economic creditors. Economic policy actions will determine the conditions for recovery, the protection of people and businesses will be essential (International Monetary Fund, 2020).

### Smart-Working and Connectivity

The total blockade at different levels- educational institutions, commercial activities, industrial production-has placed Italy facing an unprecedented challenge. In a country that has been trying to adapt to the digitalization process have emerged strongly problems related to unequal technological development, which travels at different speeds along the national territory. The problems opened up by the need to activate intelligent working methods, in all those sectors in which there is no urgent need for physical presence and/or manual work, are manifold and embrace different aspects ranging from structural issues to personal problems and needs. The digital divide essentially refers to two forms of inequality that have manifested themselves in access to adequate Internet coverage (digital infrastructure gap) and in the use of information technology (digital cultural divide).

### Digital Infrastructure Divide

The European Commission defines the digital infrastructure divide as the lack of fixed broadband coverage of at least 2 megabits. According to the latest DESI report of 2019 (The Digital Economy and Society Index), 99.5% of Italian families are served by fixed broadband, fast broadband (NGA - Next Generation Access) reaches 90% of families while only 24% it is obtained from ultrafast broadband (100 Mbps and above) (The Digital Economy Society Index, 2019). Data from the Ministry of Economic Development, referring to the last national public consultation dating back to 2017, show an even wider gap, stating that only 2% of national house numbers are reached by a connection >100 Mbps, 30% they have a connectivity higher than 30 Mbps, while almost 70% of citizens are not covered by the “ultra-wide” band (Calenda and Bentivogli, 2018). The variability of the data is dictated by the different calculation systems used but, whatever the results are closest to the real, it should be remembered that the actual navigation speed is lower than the maximum declared speed and on which these estimates are made. An emblematic example are the values of a speed test carried out by the team of University of Salerno on 17 January 2020, before therefore the coronavirus emergency, in a municipality in the province of Avellino located 725 meters above sea level, the Ospedaletto d'Alpinolo. Against a connection speed declared by the telephone operator at 20 Mbps, the actual download speed was 2.61 Mbps while the upload speed was 0.48 Mbps.

The latest Istat Citizens, Businesses and ICT (Information and Communication Technologies) report (2018) highlights a much more worrying situation. Italian families with internet access from home are 75.1%, of which 73.7% have broadband connection. In Europe, the average rate of broadband diffusion among resident households with at least one member aged 16–74 is 86%; Italy, with a rate of 83%, has a gap of 3 percentage points. Despite the growth in the number of households that have a broadband connection, the gaps still remain wide, 24.7% of households do not have access to the internet. More than one in two families declare that they do not have access to the internet at home because they do not know how to use it (58.2%), and more than one fifth (21.0%) do not consider the Internet a useful and interesting tool. There are economic reasons related to the high cost of connections or necessary tools (15.2%), while 8.1% do not surf the Net from home because at least one member of the family accesses the Internet from another place. On the other hand, the share of families who indicate insecurity with regard to the protection of their privacy (2.9%) and the lack of availability of a broadband connection (2.0%) among the reasons is residual (Istituto Nazionale di Statistica, 2019b). The main territorial gaps are found on the already known lines of inequality: north-south, city-countryside, scattered houses of urban agglomerations, coastal areas-inland areas, hilly areas-mountainous areas, continental islands.

The containment measures adopted to deal with the Covid-19 emergency have had a strong impact on the already inadequate situation of country's infrastructural network, causing an overload and a further slowdown of the same. The transition to digital does not only concern work needs but all areas of daily life, from relationships to video lessons, from recreational activities to purchases. Joy Marino director of Milano Internet Exchange, the main hub of Italian connections to and from abroad, said that in the week between 9 and 15 March there was an increase of 112% in terms of use of virtual private networks (VPN) (Levels, 2020). As is known, the problem is not limited to Italy, as of April 9, 2020 according to WHO data, 216 countries are affected by the epidemic with a total of 1,439,013 confirmed cases and 85,587 deaths (WHO, 2020), while according to when reported by AFP, the French news agency, about 4 million people are currently confined to their homes (AFP, 2020).

From late February to late March, global Internet traffic increased by 30% with an increase in bytes consumed of about 10 times compared to the average monthly data (Ruscono, 2020). In the countries affected first by Covid-19 (China, Korea, Japan, and Italy) there have been variations in Internet traffic 25% higher than the rest of the world in the same period of time, in Italy the growth in connections has been constant since beginning at the end of March (Ruscono, 2020). The Facebook CEO said that in all the countries affected by Covid-19 a very high use was made not only of WhatsApp but also of Facebook and Messenger (Levels, 2020). In Italy, the time spent on WhatsApp, Messenger and Instagram has increased by 70% since the start of the pandemic, the duration of calls on Messenger and WhatsApp has increased by more than 1,000% while the exchange of messages on these apps has increased by 50%. The evening bands are those that register the maximum increase in connection to the network up to 100% (Levels, 2020). The main risks for intelligent work are related to the overload of the so-called public cloud services, located mainly outside Italy and Europe, which could be slowed down and interrupted by limiting or completely preventing access to the sharing and communication portals, by videoconference (Levels, 2020). The main concern is related to the increase of people simultaneously in isolation and the consequent exponential growth in the use of the various platforms dedicated to relationships, leisure activities and work needs, a scenario that could lead to an inclination of the server. The minister for technological innovation, Paola Pisano, promptly reassured the country by declaring that the network will be able to resist this data overload, but has also invited to use the Internet sparingly, calling it a “precious resource” (Sky Tg24, 2020). The Italian state immediately took steps to introduce the necessary means to avoid a possible collapse of the network, art. 82 of the legislative decree 17 March 2020, n. 18 (Measures to strengthen the national health service and economic support for families, workers and businesses related to the epidemiological emergency from Covid-19)— “Cura Italia” reads “Measures intended for operators who provide communication networks and services electronics” in which operators are asked to strengthen infrastructures to guarantee the functioning of the network and the continuity of services, favoring the functioning of the health and emergency sector (Levels, 2020). The European Commission asked the main video streaming platforms to reduce the quality and speed of video playback, measures readily adopted by Netflix, Youtube and Amazon, while asking users to privilege the fixed network over the mobile network for file playback multimedia. The International Telecommunication Union (ITU) has instead implemented a “Global Network Resilience Platform” with the aim of protecting the networks of various operators during the Covid-19 crisis and supporting governments in ensuring “safer” networks and with better performance (Levels, 2020). The need to accelerate the digitization process as much as possible and to pay the utmost attention to risk management and operational continuity of the network, which is the main ally in the attempt not to restore the country's productivity and to offer a semblance of normality to everyday life or maybe build a completely different one.

### Digital Cultural Divide

The digital cultural divide highlights the division between the part of the population with digital skills from others who does not possess these skills. Digital exclusion seems to follow the already known lines of social discrimination, which affect the elderly, unemployed or women in particular conditions, immigrants, people with disabilities, prisoners and all those with low levels of education and training. The 2019 DESI report highlights the sharp gap between Italy and the rest of the European Union countries in terms of human capital. Italy ranks 26th with an average of 32.6% compared to the EU average of 48%, only 44% of people between 16 and 74 have basic digital skills while the percentage is even lower than people who possess digital skills higher than the basic ones equal to 19% (The Digital Economy Society Index, 2019). Even more worrying is the figure of the habitual use of the Internet by young people between 16 and 24 years of age, which sees Italy in last place among the 28 EU member countries with a percentage of 92% compared to 97 Average% of the EU (The Digital Economy Society Index, 2019). From 9 March the activities of educational institutions were suspended until a later date, the executive immediately recommended the implementation of distance learning to make it mandatory, with the law decree of 8 April 2020, n. 22 (Urgent measures on the regular conclusion and the orderly start of the school year and on the conduct of state exams) approved by the Council of Ministers, Monday 6 April.

The country has responded with different times and ways starting from irregular starting resources. As highlighted by the DESI 2019 report, the National Plan for digital school, launched in Italy in 2015, does not seem to have produced important results, only 20% of teachers have attended digital literacy courses and 24% of schools do not yet have courses of programming (The Digital Economy Society Index, 2019). On the same day that the government approved compulsory distance learning, ISTAT released a note entitled “Home spaces and availability of computers for children and adolescents” which highlights the difficulty situation in a country where inequalities continue to be high, especially along the north-south axis. Based on the data collected in the years 2018–2019, it appears that 33.8% of families do not have a computer or tablet at home, a percentage that drops to 14.3% among families with at least one minor and still decreases reaching 7.7% in families where at least one component has a degree, clarifying how the level of education weighs on the digital cultural divide (Istituto Nazionale di Statistica, 2020a,b,c). In the south the percentage of families without computers exceeds 41% compared to 30% in other areas of the country, the same gap occurs for the number of computers in homes in relation to the number of family members, in the south 26, 6% of Households has a number of PCs and tablets available for less than half of the components and only 14.1% have at least one for each component (Istituto Nazionale di Statistica, 2020a,b,c). Fifty seven percentage of young people aged between 6 and 17 must share a computer or tablet with the family, this implies that even in cases where Internet access is present (96% of families) this does not guarantee the possibility to carry out distance learning also considering that this is generally the students at the same time as adults are engaged in intelligent work (Istituto Nazionale di Statistica, 2020a,b,c). In addition, a good level of connection is required to follow the online lessons, which prevents audio from skipping or blocking the video, Infodata has reworked ISTAT data (Aspects of daily life 2019) showing that families with a band connection wide fixed are two out of three in Lazio (figure > 62.2%) while they do not exceed 41% in Calabria and Basilicata with a greater penalty for residents of small municipalities (Orlando, 2020; Saporiti, 2020). As for digital skills, according to ISTAT data, only 30.2% of young people between 14 and 17 years of age have high digital skills, 3% do not have digital skills while about two thirds have low or base (Istituto Nazionale di Statistica, 2020a,b,c). The quality of work and home study are also strongly influenced by another important factor, housing conditions. According to ISTAT data from 2018, 27.8% of people in Italy live in conditions of housing overcrowding, a difficult condition experienced in particular by minors with a percentage of 41.9% living in conditions of overcrowding. A discomfort that is aggravated in the presence of structural housing problems such as cramped, poorly ventilated and poorly lit spaces, what Istat defines as serious housing deprivation concerns 5% of Italians, also in this case young people, 7.0% of minors and 7.9% of young people aged between 18 and 24 live in conditions of housing discomfort (Istituto Nazionale di Statistica, 2020a,b,c).

### Social Distancing and Empathy

The response to the Covid-19 pandemic is infiltrating every aspect of life, social distance has generated serious consequences that are already being felt. The struggle against this invisible enemy could last for months or even years. Public health experts believe that social distancing is the best way to prevent a truly horrible crisis, beyond a certain threshold the national health system is unable to accept and treat people who require fans and intensive care units. To date, the only measure that seems to be working is the strict Social Distance policy. The elimination of these measures could now trigger new outbreaks that would seriously jeopardize public health. It is not possible to predict when the virus disappears and restrictive measures will continue for as long as necessary, seriously affecting social relationships and interactions, especially the empathic process. Empathy refers to the ability to put oneself in another person's situation or, more precisely, to understand the other person's emotional processes and respond in a congruent way. This term means a German term in Italian, Einfühlung (Treccani, 2019b). This term is placed at the base of the aesthetic theory elaborated by Vischer (1847–1933) and Lipps, according to whom art is the identification of feeling in natural forms, thanks to a deep consonance or sympathy between subject and object (Vischer and Vischer, 1887; Lipps, 1903). The individual attributes beauty to the forms in which he manages to transfer or project his vital sense. Aesthetic enjoyment is therefore objectified enjoyment of ourselves (Treccani, 2019a,b,c). Starting from the early 90s the problem of understanding empathy, understood as that form of identification in the psychological states, which more and more often fall into physiological states, of the other to which the explanation, or understanding, of his behavior would be subordinated at the center of a meaningful and lively debate in the philosophy of psychology and in the philosophy of the mind, which today falls within the cognitive sciences (Franks, 2010). Without prejudice to the reference to the historical models of empathic understanding by Dilthey (1833–1911), such as Verstehen by Weber (1864–1920), Schutz (1899–1959), Simmel (1858–1918) and the re-enactment of Collingwood (1889–1943), the renewed debate began with some developments in the analytical philosophy of language and mind, in particular with a thesis by Quine (1908–2000).

According to Quine, the attribution of the so-called propositional attitudes or intentional states, through which the psychology of common sense normally explains the behavior of individuals according according to the classic model of purpose of the means, is essentially based on an empathic simulation (Treccani, 2019a,b,c). This empathic simulation constitutes, for Quine, a natural epistemic modality with which beliefs, desires and perceptions are currently, and often unconsciously, attributed (Quine, 1990, 1992). Trying to analyze this aspect within the dynamics of life, it can be said that a “lowering of empathy levels” is very likely to occur. Although, on the one hand, there may be known cases, reported every day by newspapers and television news, in which one or more people identify with those who suffer, as neighbors, friends, relatives or with those who live complex experiences, situations and disadvantages from the point of view. In view of health and finance, they promote activities such as so-called “suspended expenses.” Solidarity activities such as humanitarian aid of any kind or even the simple home delivery of food and medicine for people who do not have the opportunity to go out and meet their needs increase. However, there is a slice of the population that does as empirical evidence in which it has been addressed that physical and psychological distance can modulate the empathic reaction of a person who is observing someone else in pain. So being further away makes the empathic reaction less strong. I think this point is crucial, as this block has forced people to keep their distance, creating a scenario where no one can be close to others and/or you have to wear a mask, which prevents you from feeling the same “level of empathy.” However, there is a part of the population that suffers, more than the other, the current situation (social distance, mask, etc.). In this regard, an extremely interesting research by Lomoriello et al. (2018), through empirical evidence, that physical and psychological distance can modulate the empathic reaction of a person who is observing someone else in pain. So being further away makes the empathic reaction less strong. The reference is clearly addressed to the technology that is used, which, even in this phase, depersonalizes relationships on the one hand, or rather distorts them as forced to use them, but brings us closer, albeit virtually, to the other. Humanity finds itself experiencing a kind of hyperconnected feeling in an attempt to mitigate the blow it took when the forced “fence” was declared.

“This evidence provides an important insight into the framework of knowledge on factors capable of shaping empathy, and it is certainly important also in relation to the evidence suggesting a strong link between representations, also in neural terms, of physical and psychological distance. Although it is obvious that in everyday life situations it is not possible to establish in advance the physical distance between an observer and someone subjected to physical pain (given the unpredictability of such situations), the evidence on the importance of physical distance in modulating an empathic reaction could be fundamental for psychotherapy, clinical and medical contexts, in which psychotherapists, doctors and health professionals could use this knowledge to favor or not, as appropriate, an empathic reaction in themselves and in their patients” (Lomoriello et al., 2018, p. 11).

In conclusion, therefore, a strong forcing that could have the effect of withdrawing completely from the other, once the whole virus situation is over. In the worst case, you could see the intensification of some social phobias, the same ones that had previously been alleviated thanks to a simple meeting in the office or on the street. In this moment, even the “how are you” at the beginning of a phone call, video call or conference call is no longer just a formality, as it once was. For example, intelligent workers, discussed in detail in the previous paragraphs, now, when they call their collaborators, must expect after the usual question, not a simple “good,” but much more complex answers. Generating in those who receive the request the false belief that the other is actually interested in their situation. While those who pose it could generate anxiety and anguish given the obligation to listen, feeling almost obliged, to the answers that the other person is giving. Thus, it can be thought that the situation of the aforementioned “forced empathy” may occur. This is because the distance and the emergency situation make people want to be heard and appreciate “how are you?” the hook to express fears, emotions, fears and weak points (Pasetti, 2020).

## Discussions

Each nation is facing the emergency from a different starting condition that will inevitably affect the timing and methods of recovery.

It is not yet possible to predict if, how and when Italy will rise, but what is not hoped for is a return to normality. That normality in which every day individuals have to fight to improve their conditions, of life and work, and to ask, at most, when they do not have to defend those who have been conquered by centuries of social struggles, for greater rights. The crises faced so far and the recipes adopted to overcome them do not lead us to hope to be able to build a better, more just, more favorable, less unequal world, but once again to see wages decrease, precariousness increase, go backwards, poverty and unemployment reach historic highs. This time it will not be the army that will stop the cry of despair that rises from the world, but an invisible threat that stands between individuals and keeps them separate, everyone will be more committed to defending themselves from the other and they do not have the strength to defend themselves together. It is all that humanity does not hope for.

## Conclusion

To conclude, referring above all to the latest developments with the OpenFiber agreement in Italy, the political question underlying the problems due to connectivity is of long standing. Even today we can see the duality of Italy, on the one hand the one characterized by super-speed and hyper-connection, on the other the one that has network infrastructural deficiencies (total absence of connection) and that struggles to have a stable connection. All this translates into an economic delay for the entire country, mainly due to the lack of growth opportunities that today, more and more, pass through the network. With the hope that the OpenFiber project, approved by the government, can really solve one of the biggest problems and that this can slightly reduce the gap with the whole of Europe. Furthermore, the Covid-19 crisis phase has brought out with greater force the work problems already present in previous years. In particular, following the lockdown phase, it emerged that the continuous cuts made to funds destined for universities, research and healthcare, have led to a structural and organic deficiency that has generated deep micro-crises within the crisis itself. Therefore, the hope is that Covid-19 will serve as a lesson and that more funds will be allocated to sectors that are objectively fundamental for society.

## Author Contributions

VA and CI conceptualized the contribution. VA wrote the paper. CI reviewed the manuscript and provided the critical revision processes as PI. All authors approved the submission of the manuscript.

## Conflict of Interest

The authors declare that the research was conducted in the absence of any commercial or financial relationships that could be construed as a potential conflict of interest.

## Footnotes

^1^The obligation to wear a mask has not been introduced uniformly on the national territory. This obligation was mostly provided for in a “transversal” way, that is only for certain population groups (health professionals, commercial operators of basic necessities such as food, pharmacies, etc.) and, in any case, only at the regional level.
